# The Role of Sirt6 in Obesity and Diabetes

**DOI:** 10.3389/fphys.2018.00135

**Published:** 2018-02-27

**Authors:** Jiangying Kuang, Lei Chen, Qin Tang, Jinhang Zhang, Yanping Li, Jinhan He

**Affiliations:** ^1^State Key Laboratory of Biotherapy, Department of Pharmacy, West China Hospital, Sichuan University, Chengdu, China; ^2^Department of Cardiology, The Second Hospital of Shandong University, Shandong University, Jinan, China; ^3^Laboratory of Clinical Pharmacy and Adverse Drug Reaction, West China Hospital, Sichuan University, Chengdu, China

**Keywords:** Sirt6, obesity, diabetes mellitus, type 2, LiPo, gluconeogenesis

## Abstract

Sirt6 is one of the sirtuin family members, a kind of NAD+-dependent histone deacetylase and ADP-ribose transferase enzyme. It has an important role in physiological and pathological processes, regulating aging, cancer, obesity, insulin resistance, inflammation, and energy metabolism. Recent studies have suggested that reduced Sirt6 action is related to obesity and diabetes. Aging and overnutrition, two major risk factors for obesity and diabetes, lead to decreased Sirt6 level and function, which results in abnormal glucose and lipid metabolism. Whole-body ablation of Sirt6 in mice results in severe hypoglycemia. Sirt6 deficiency leads to liver steatosis and promotes diet-induced obesity and insulin resistance. Sirt6 has a protective effect on obesity and diabetes. This review surveys evidence for an emerging role of Sirt6 as a regulator of metabolism in mammals and summarizes its major functions in obesity and diabetes.

## Introduction

The sirtuins are a highly conserved family of NAD^+^-dependent deacetylases and ADP-ribosyltransferases that play an important regulatory role in the physiological and pathological processes of the organism. They participate in regulating the life span and aging, cancer, obesity, insulin resistance, inflammatory response and energy metabolism (Michan and Sinclair, 2007). The founding member of the sirtuin family, Sirt2, was originally discovered in *Saccharomyces cerevisiae*. Currently, seven sirtuins (Sirt1-7) have been found in mammals, each containing the conserved sirtuin core domain that confers NAD^+^-dependent deacetylase activity (Frye, 1999).

Each member has distinct subcellular localizations, targets and functions. Sirt1 and Sirt2 were found in both the nucleus and cytoplasm; Sirt3, Sirt4, and Sirt5 were found in mitochondria, and Sirt6 and Sirt7 were found in the nucleus (Michishita et al., 2005). Sirt1-3 have strong deacetylase activity, whereas Sirt4 has ADP-ribosyltransferase activity (Michishita et al., 2005). Sirt5 has weak NAD^+^-dependent deacetylase, desuccinylase and demalonylase activity (Michishita et al., 2005). Sirt7 was recently shown to be a highly specific deacetylase (Michishita et al., 2005). Sirt6 has several enzymatic activities, including NAD^+^-dependent deacetylase activity and mono-ADP-ribosyl transferase activity of acetyl groups and long-chain acyl groups. The most well-studied sirtuin is Sirt1, and the study of Sirt6 is still in its infancy.

As one of NAD^+^-dependent deacetylases, Sirt6 was first cloned from a human spleen cDNA library (Liszt et al., 2005). Recent studies have suggested reduced Sirt6 activity related to obesity and diabetes. Aging and overnutrition, two major risk factors for obesity and diabetes, lead to decreased Sirt6 level and function and result in abnormal glucose and lipid metabolism. Whole-body ablation of Sirt6 in mice resulted in severe hypoglycemia (Kanfi et al., 2008, 2010; Kuang et al., 2017; Yao et al., 2017). Hepatic-specific ablation of Sirt6 increased liver steatosis (Kim et al., 2010). Fat-specific deletion of Sirt6 increased blood glucose levels and hepatic steatosis and promoted diet-induced obesity and insulin resistance (Kuang et al., 2017; Xiong et al., 2017; Yao et al., 2017). Neural-specific deletion of Sirt6 in mice promoted diet-induced obesity and insulin resistance (Schwer et al., 2010). In contrast, Sirt6 overexpression protected against diet-induced obesity and insulin resistance (Kanfi et al., 2010).

These recent studies showed that Sirt6 plays an important role in lipid and glucose metabolism. This review summarizes the role of Sirt6 in obesity and diabetes.

## Sirt6 and glucose metabolism

**Figure 1:**
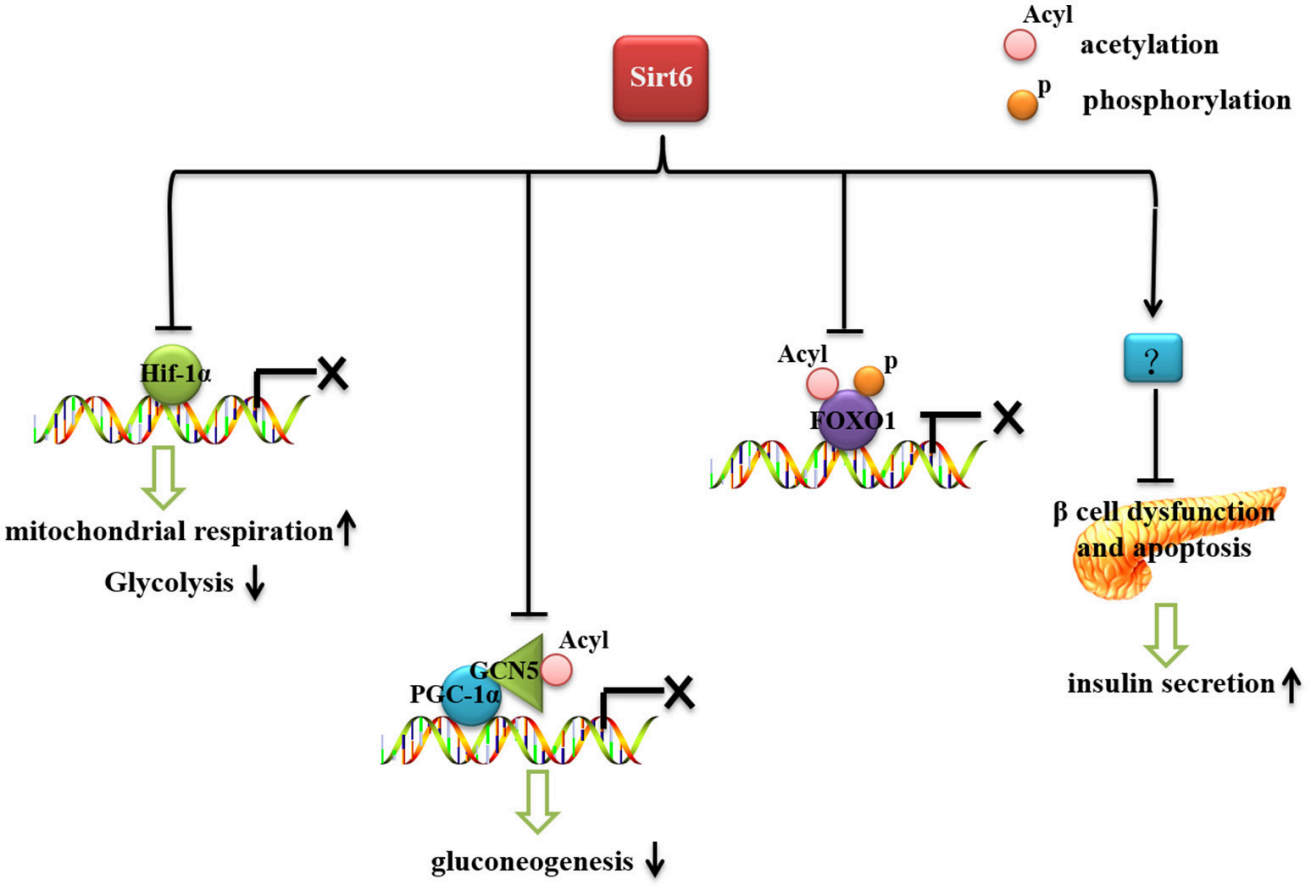
The role of Sirt6 in glucose metabolism. Sirt6 inhibits recruitment of Hif-1α to its target gene promoters, increases mitochondrial respiration and inhibits glycolysis. Sirt6 binds to and activates GCN5, inhibits the acetylation of PGC-1α, and decreases the expression of gluconeogenic genes. Sirt6 can specifically interact with FoxO1, decrease FoxO1 acetylation and phosphorylation level, and inhibit the interaction between FoxO1 and its downstream gene promoters, thereby reducing the expression of gluconeogenic genes. Sirt6 protects against β-cell dysfunction and apoptosis and increases insulin secretion.

Glucose is an essential energy source needed by all cells and organs of our bodies. Therefore, blood glucose should be kept at a certain level to maintain tissue and organ energy requirements. The imbalance of glucose homeostasis is an important pathogenic factor of diabetes, obesity and other metabolic diseases. Sirt6 plays an important role in glucose production and metabolism (Figure 1). Mice with whole-body Sirt6 deficiency showed severe hypoglycemia (Mostoslavsky et al., 2006). Subsequent studies found that Sirt6 affects both gluconeogenesis and glycolysis (Zhong et al., 2010; Dominy et al., 2012; Zhang et al., 2014). Moreover, recent reports suggested that Sirt6 regulates pancreatic β-cell function, an important organ for maintaining blood glucose level (Kugel et al., 2016; Demir et al., 2017).

### Sirt6 and blood glucose

The first clue that Sirt6 might play a role in glucose metabolism came from a severe hypoglycemia phenotype observed in Sirt6-deficient mice (Mostoslavsky et al., 2006). Mice with whole-body Sirt6 deficiency showed increased glucose uptake and enhanced insulin signaling (Xiao et al., 2010). Sirt6-deficient mice, although small, appear normal in the first 2 weeks, then show a series of acute degenerative phenotypes, dying at about age 1 month (Mostoslavsky et al., 2006). The phenotypes include lymphopenia, osteopenia, lordokyphosis and loss of subcutaneous fat (Mostoslavsky et al., 2006). The most striking phenotype is severe hypoglycemia, which could be the main factor killing the mice before age 1 month. However, the severe hypoglycemia phenotype may be not triggered by the abnormal insulin level, because blood insulin levels are even lower than in wild-type mice (Xiao et al., 2010). When the mice were fed water containing 10% glucose, blood glucose was increased and about 83% of the mice with whole-body Sirt6 deficiency survived, indicating the hypoglycemia was a main factor in the postnatal lethality (Xiao et al., 2010).

Xiao et al. found that Sirt6 negatively regulates Akt phosphorylation at Thr 308 and Ser 473 by inhibiting multiple upstream molecules, insulin receptors, and insulin receptor substrate 1 and 2 (Xiao et al., 2010). With Sirt6 deficiency, insulin signaling is activated and Akt phosphorylation is increased (Xiao et al., 2010). Then, glucose uptake is increased, which may cause hypoglycemia. In contrast, Sirt6 deficiency does not affect intestinal glucose absorption and does not lead to higher glucose secretion in the kidney (Zhong et al., 2010). *In vivo* study by ^18^F-fluorodeoxyglucose-positron emission tomography (FDG-PET) in Sirt6-deficient mice showed increased glucose uptake in both brown adipose tissue and muscle but not liver, brain or heart, which could further explain the hypoglycemic phenotype (Zhong et al., 2010). The increased glucose uptake in these tissues could be explained by higher expression of glucose transporter 1 (Glut1), one of main glucose transporters that modulates basal uptake of glucose, independent of insulin or growth factor (Zhong et al., 2010). The central nervous system also plays an important role in regulating glucose metabolism. Growth hormone and insulin-like growth factor 1 (IGF-1) levels were lower in mice with brain-specific Sirt6 knockout than control mice, similar to mice with whole-body Sirt6 knockout (Schwer et al., 2010). Thus, under certain physiological conditions, Sirt6 may affect glucose metabolism and insulin sensitivity via growth hormone/IGF-1 signaling.

### Sirt6 and glycolysis

With sufficient oxygen, glucose is metabolized to pyruvate, which is further converted to ATP in mitochondria. However, in the absence of nutrients or during hypoxia, cells undergo anaerobic respiration and pyruvate is converted to lactic acid (Aragonés et al., 2009; Vander Heiden et al., 2009). In understanding the hypoglycemia seen in Sirt6-deficient mice, Zhong et al. demonstrated that Sirt6 regulates glucose homoeostasis by suppressing the expression of multiple glycolytic genes (Zhong et al., 2010). This suppression results in efficient ATP production via mitochondrial oxidative phosphorylation instead of glycolysis. Loss of Sirt6 increases glycolysis and diminishes mitochondrial respiration (Aragonés et al., 2009). The role of Sirt6 in glycolysis is mediated by hypoxia-inducible factor 1α (Hif-1α), known to regulate glycolysis and mitochondrial respiration in a coordinated manner (Zhong et al., 2010). Sirt6 deficiency induced Hif-1α activity and then increased the expression of glycolysis-related genes such as Glut1, lactate dehydrogenase (LDH), phosphoglycerate kinase (PGK1), glucose-6-phosphate isomerase (GPI), and phosphofructokinase 1 (PFK-1), and promoted glycolysis (Hu et al., 2006; Zhong et al., 2010). Simultaneously, activated Hif-1α directly inhibited mitochondrial respiration by increasing the expression of dehydrogenase kinase (PDK) (Kim et al., 2006; Papandreou et al., 2006). Moreover, when mice with Sirt6 deficiency were treated with an Hif-1α inhibitor, the hypoglycemia phenotype was rescued, which suggests that increased activity of Hif-1α contributes to the impaired glucose metabolism in these mice (Zhong et al., 2010).

Further study revealed that Sirt6 could regulate Hif-1α via two plausible scenarios: (1) Sirt6 inhibits recruitment of Hif-1α (accelerating its degradation) to its target gene promoters, or (2) Hif-1α could already localize to the promoters under normoglycemia, but the presence of Sirt6 would inhibit its transcriptional activity (Zhong et al., 2010).

### Sirt6 and gluconeogenesis

In addition to regulating glycolysis, Sirt6 affects gluconeogenesis. In the absence of Sirt6, hepatic gluconeogenesis was significantly elevated, which suggests a compensatory response to hypoglycemia (Dominy et al., 2012). Gluconeogenesis is tightly controlled by various cellular signaling pathways and transcription factors (Magnusson et al., 1992). Peroxisome proliferator-activated receptor γ coactivator 1-α (PGC-1α) is a key transcriptional regulator for gluconeogenesis. PGC-1α increases the expression of gluconeogenic enzymes such as glucose-6-phosphatase (G6p) and phosphoenolpyruvate carboxykinase (Pepck) (Puigserver et al., 2003). The transcriptional activity of PGC-1α is negatively regulated by its acetylation level. General control non-repressed protein 5 (GCN5) increased the acetylation level of PGC-1α and decreased PGC-1α transcriptional activity (Lerin et al., 2006). Sirt6 could directly bind to and activate GCN5 (Dominy et al., 2012). With knockout of Sirt6, GCN5 activity is decreased, the acetylation level of PGC-1α is reduced and PGC-1α controls the expression of gluconeogenic genes (Dominy et al., 2012).

Forkhead box protein O1 (FoxO1) also plays an important role in regulating gluconeogenesis. FoxO1 activates gluconeogenesis by directly binding the promoter regions of G6p and Pepck (Schilling et al., 2006). With mutation of the FoxO1 transcriptional activation domain and activity abolished, gluconeogenesis was significantly diminished (Nakae et al., 2001). FoxO1 deficiency significantly impaired the fasting-induced expression of G6p and Pepck (Matsumoto et al., 2007). The transcriptional activity of FoxO1 is mainly regulated by its phosphorylation and acetylation (Brunet et al., 2004; Yamagata et al., 2008; Zhao et al., 2010). In Sirt6-deleted cardiomyocytes, FoxO1 phosphorylation was increased (Sundaresan et al., 2012). The phosphorylation of FoxO1 promotes the translocation of FoxO1 from the nucleus to the cytoplasm, thereby reducing its transcriptional activity. Subsequent studies found that Sirt6 can specifically interact with FoxO1, thereby inhibiting the interaction between FoxO1 and its downstream genes G6p and Pepck, to reduce the expression of gluconeogenic genes (Zhang et al., 2014).

### Sirt6 and pancreatic β-cell function

The connection between Sirt6 and glucose metabolism is strengthened by the critical role of Sirt6 in promoting glucose-stimulated insulin secretion and ATP production in pancreatic β-cells (Xiong et al., 2016). These effects might be related to evidence of mitochondria damage (mitochondrial function and Ca^2+^ dynamic regulation in β cells impaired in Sirt6-deficient mice) and lower rate of oxygen consumption seen in Sirt6-deficient pancreatic β cells (Xiong et al., 2016). Sirt6 ablation also increases cell apoptosis and impairs insulin secretion in response to glucose in MIN6 cells (β-cell lines). Conversely, Sirt6 overexpression protects against palmitate-induced β-cell dysfunction and apoptosis (Song et al., 2016; Xiong et al., 2016). Recently, a new study found that Sirt6 is critical for pancreatic β-cell function and survival in mice (Qin et al., 2018). Sirt6 deficiency does not affect endocrine morphology, β-cell mass or insulin production but did result in glucose intolerance and defective glucose-stimulated insulin secretion in mice (Qin et al., 2018). β-cell specific deletion of Sirt6 reproduced the defect in insulin secretion. Loss of Sirt6 increases acetylation of histone H3K9Ac, H3K56Ac, and activates RNA polymerase II at the promoter region of thioredoxin-interacting protein (TXNIP) (Qin et al., 2018). TXNIP expression is negatively associated with glucose-stimulated insulin secretion in β-cells and that overexpression of TXNIP inhibits insulin secretion (Rani et al., 2010; Yoshihara et al., 2010; Luo et al., 2014). Sirt6-deficiency in β-cells exhibited a time-dependent increase in H3K9Ac, H3K56Ac, and TXNIP levels. Finally, β-cell specific Sirt6-deficient mice showed increased sensitivity to streptozotocin induced β-cell apoptosis (Qin et al., 2018). Together, this report indicates that Sirt6 has a key role in pancreatic function.

## Sirt6 and calorie restriction

Calorie restriction (CR) reduces cellular NADH concentration, thereby increasing the NAD+/NADH ratio and promoting Sirt2 activity (Tasselli et al., 2016a). Sirt6 activity is significantly modulated by CR. It is increased by nutrient depletion or long-term CR in the brain, white adipose tissue (WAT), muscle, liver and kidney in mice (Kanfi et al., 2008; Kuang et al., 2017). Sirt6 also mediates the effects of CR, which is known to delay the onset of age-associated diseases, including diabetes and cardiovascular diseases. Sirt6 ablation abolished CR-induced life extension. Moreover, CR-activated Sirt6 suppressed NF-κB signaling and delayed aging (Zhang et al., 2013). Transgenic mice overexpressing Sirt6 showed multiple phenotypes resembling CR, including reduced body weight, enhanced metabolic activity, and reduced serum levels of cholesterol, adipokines, insulin, and glucose, which further demonstrates the regulatory role of Sirt6 in energy metabolism (Kanfi et al., 2010, 2012).

## Sirt6 and lipid metabolism

**Figure 2:**
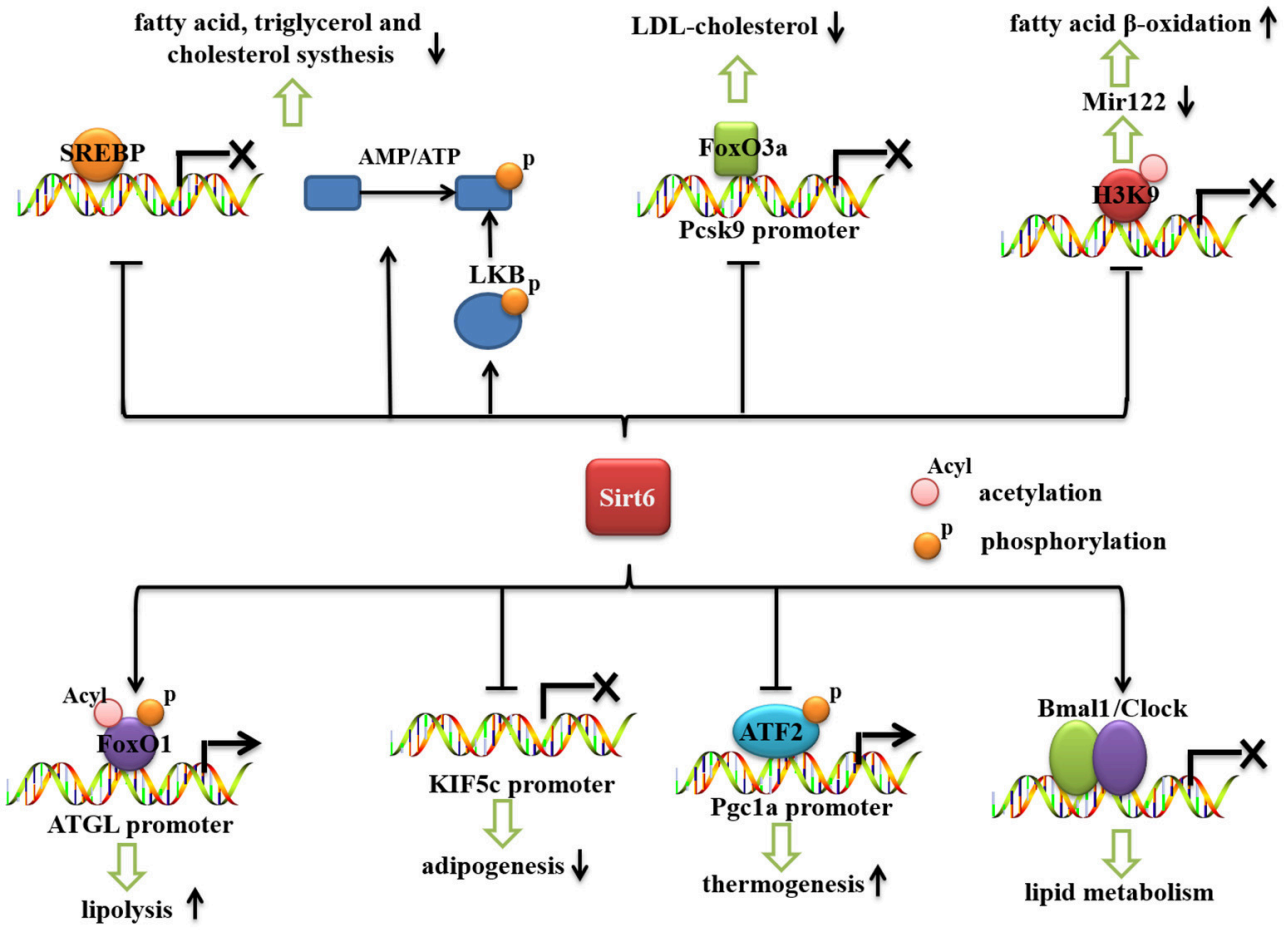
The role of Sirt6 in lipid metabolism. Sirt6 represses the mRNA expression of SREBP1/2 and inhibits the cleavage of SREBPs into their active forms. Sirt6 activates AMPK (by increasing the AMP/ATP ratio and activating LKB), which phosphorylates and inactivates SREBP. Sirt6 can be recruited by FoxO3a to the Pcsk9 promoter, suppress its gene expression and decrease the level of LDL-cholesterol. Sirt6 downregulates miR-122 by deacetylating H3K56, reduces miR-122 expression, and increases fatty acid β-oxidation. Sirt6 also specifically interacts with FoxO1, decreases FoxO1 acetylation and phosphorylation, and promotes the interaction between FoxO1 and the promoter of its downstream gene, ATGL, thereby increasing ATGL expression and inducing lipolysis in adipose tissue. Sirt6 inhibits KIF5C gene expression and impairs adipogenesis. Sirt6 regulates phospho-ATF2 (p-ATF2) binding to the PGC-1α promoter and subsequently affects the thermogenic program. Sirt6 regulates the circadian rhythm in the liver by maintaining a proper cyclic recruitment of CLOCK/BMAL1.

Recent reports have shown that Sirt6 is an important regulator of lipid metabolism (Figure 2). Sirt6 regulates the hepatic accumulation of triglycerides (TG), which is associated with fatty liver disease (Kugel and Mostoslavsky, 2014; Vitiello et al., 2017). Sirt6 deficiency promotes lipogenesis and fatty acid uptake but inhibits β-oxidation (Kim et al., 2010). Cholesterol synthesis is also negatively regulated by Sirt6 (Elhanati et al., 2013). Sirt6 positively regulates lipid mobilization and thermogenesis in adipose tissue (Chen et al., 2017; Kuang et al., 2017; Xiong et al., 2017; Yao et al., 2017). Moreover, Sirt6 regulates circadian metabolic programs, accompanied by changes in lipid metabolism (Masri et al., 2014).

### Sirt6 and hepatic lipid metabolism

The liver is a key metabolic organ controlling aspects of lipid metabolism in response to hormonal or nutritional signals. Under starvation, the liver converts lipid stores into available energy via fatty acid oxidation. In the fed condition, metabolic programs in the liver are switched on to store energy in the form of lipid droplets via the process of lipogenesis (Puigserver et al., 2003).

In addition to its critical regulatory role in glucose metabolism, Sirt6 plays an important regulatory role in hepatic lipid metabolism. Hepatic-specific disruption of Sirt6 in mice resulted in fatty liver formation (Kim et al., 2010). Hepatic TG accumulation in liver is mainly regulated by fatty acid uptake, β-oxidation, and TG synthesis and secretion. Deletion of Sirt6 increased the expression of hepatic fatty acid transport genes and reduced that of fatty acid β-oxidation genes (carnitine palmitoyltransferase 1 [CPT1α], acyl-coenzyme A oxidase 1 [ACOX1]). Genetic deletion of Sirt6 in mice increased the mRNA levels of lipogenic genes such as acetyl CoA carboxylase 1 (ACC1), fatty acid synthase (FAS), and stearoyl-CoA desaturase-1 (SCD1) (Kim et al., 2010).

Rosiglitazone (RGZ), an agonist of peroxisome proliferator-activated receptor gamma (PPARγ), plays an important role in protecting against fatty liver disease. Activation of PPARγ by RGZ increased Sirt6 expression (Yang et al., 2011). RGZ treatment ameliorated hepatic lipid accumulation and increased the expression of Sirt6, PGC-1α, and FoxO1 in rat liver (Yang et al., 2011). In addition, AMP-activated protein kinase (AMPK) phosphorylation was increased by RGZ. Sirt6 knockdown increased hepatocyte lipid accumulation and abolished the effect of RGZ on hepatic steatosis (Yang et al., 2011). Sirt6 knockdown also abolished the effect of RGZ on the mRNA and protein expression of PGC1-α and FoxO1 and phosphorylation levels of AMPK, which suggests that Sirt6 is involved in the RGZ-mediated metabolic effects (Yang et al., 2011). However, this result contradicts a report that Sirt6 inhibits the activity of PGC-1a by increasing GCN5 expression (Dominy et al., 2012), so Sirt6 may have different regulatory roles in different models. MicroRNA-122 (miR-122), a microRNA (miRNA) highly expressed in liver, constitutes 70% of the total miRNA pool in liver (Chang et al., 2004; Bhattacharyya et al., 2006; Jopling, 2012). MiR-122 plays an important regulatory role in many metabolic processes, including cholesterol synthesis and fatty acid oxidation (Krützfeldt et al., 2005; Esau et al., 2006). Sirt6 and miR-122 are reciprocally regulated to control the gene expression of fatty acid oxidation (Elhanati et al., 2016). Sirt6 downregulates miR-122 by deacetylating H3K56. MiR-122 binds to three sites on the Sirt6 3' untranslated region and reduces its levels. Overexpression of Sirt6 in mouse liver reduced miR-122 expression and increased that of fatty acid β-oxidation genes (Elhanati et al., 2016).

Sirt6 also regulates low-density lipoprotein (LDL)-cholesterol levels (Kanfi et al., 2010). Sirt6 regulates cholesterol level via the lipogenic transcription factors sterol regulatory element binding proteins 1/2 (SREBP1/2) and AMPK (Elhanati et al., 2013). It represses the expression of SREBP1/2 and by three mechanisms: it represses the transcription levels of SREBP1/2 and inhibits their cleavage into their active forms (Elhanati et al., 2013) and it activates AMPK (by increasing the AMP/ATP ratio and activating LKB), which phosphorylates and inactivates SREBP1 (Elhanati et al., 2013). Proprotein convertase subtilisin/kexin type 9 (PCSK9) is a crucial gene regulating LDL-cholesterol (Tao et al., 2013). Sirt6 plays a critical role in regulating Pcsk9 gene expression. Sirt6 can be recruited by FoxO3a to the Pcsk9 gene promoter and deacetylate histone H3 at Lys 9 and 56, thereby suppressing the gene expression (Tao et al., 2013). Conversely, overexpression of Sirt6 decreased LDL-cholesterol level in high-fat diet (HFD)-fed mice (Kanfi et al., 2010).

### Sirt6 and adipose tissue lipolysis

Sirt6 regulates the lipid metabolism of adipose tissues. Sirt6 ablation mediated by ap2-CRE or adiponectin-CRE in adipose tissue increased HFD-induced obesity and insulin resistance (Kuang et al., 2017; Yao et al., 2017), and Sirt6 overexpression inhibited HFD-induced obesity and insulin resistance (Kanfi et al., 2010). Sirt6 ablation increased diet-induced obesity via adipocyte hypertrophy rather than abnormal adipocyte differentiation (Kuang et al., 2017). Adipocyte hypertrophy in Sirt6-deficient mice might be attributed to impaired lipolytic activity, which causes fat storage synthesis exceeding lipolysis and results in obesity. Adipose TG lipase (ATGL) is the key lipase that hydrolyzes TG into diglycerides (Zimmermann et al., 2004; Kuang et al., 2017); Sirt6 ablation suppressed ATGL expression. Sirt6 physically interacts with FoxO1, and Sirt6 deficiency increased the acetylation and phosphorylation of FoxO1, thereby promoting its nuclear exclusion and decreasing its transcriptional activity, which downregulated ATGL expression (Kuang et al., 2017). The role of Sirt6 in regulating FoxO1 is contradictory in liver and adipose tissue (Zhang et al., 2014; Kuang et al., 2017), which indicates that the regulatory function of Sirt6 is tissue-specific. Sirt6 overexpression protects against HFD-induced physiological damage by blocking the lipotoxicity of obesity and restoring glucose homeostasis via specific reduction of PPARγ signaling and level of diacylglycerol acyltransferase 1 (DGAT1), a key regulator of TG synthesis (Kanfi et al., 2010).

### Sirt6 and adipogenesis

Adipose tissue, differentiated from pre-adipocytes, is regulated by multiple transcriptional factors, including PPARγ and CCAAT/enhancer-binding protein α, β, and δ (C/EBPα/β/δ). Recently, Chen et al found that Sirt6 deficiency in preadipocytes blocks their adipogenesis (Chen et al., 2017). Sirt6 deficiency impairs adipogenesis, and Sirt6 is an essential factor for mitotic clonal expansion during adipogenesis by inhibiting the expression of kinesin family member 5C (KIF5C) and enhancing casein kinase 2 (CK2) activity. KIF5C is negatively regulated by Sirt6. Moreover, this study showed that KIF5C is a negative factor for adipogenesis by interacting with CK2a, a catalytic subunit of CK2 (Chen et al., 2017).

### Sirt6 and thermogenesis

Obesity is due to a chronic imbalance between energy intake and energy expenditure. WAT is essential for TG storage and insulin resistance, whereas brown adipose tissue (BAT) generates heat by dissipating energy via uncoupled respiration mediated by uncoupling protein 1 (UCP1) (Lowell and Spiegelman, 2000; Nedergaard and Cannon, 2010). WAT could be converted into brown-like adipocytes (beige cells). Cells undergoing a browning process have been suggested to have strong anti-diabetic or anti-obesity benefit (Cypess et al., 2009; Barbatelli et al., 2010; Petrovic et al., 2010; Wu et al., 2012). Recent studies show that Sirt6 has a critical role in regulating the thermogenesis of fat (Yao et al., 2017). Cold exposure and a β-adrenergic agonist markedly induced Sirt6 expression in fat. Fat-specific ablation of Sirt6 mediated by Ap2-CRE impairs the thermogenic function of brown adipocytes, thereby causing a morphological “whitening” of brown fat, and decreased oxygen consumption, core body temperature and cold sensitivity (Yao et al., 2017). PGC-1α is highly expressed in BAT and is a central regulator of brown fat thermogenesis (Puigserver et al., 1998). White fat cells overexpressing PGC-1α show mitochondrial oxidation phosphorylation and expression of thermogenesis genes (Puigserver et al., 1998). Sirt6 depletion markedly decreased the expression of PGC-1α and other thermogenic genes (Yao et al., 2017). Sirt6-depleted adipocytes also decreased basal mitochondrial respiration and maximal mitochondrial respiratory capacity. Mitochondrial oxidative phosphorylation and the expression of biogenesis genes are decreased significantly in primary brown adipocytes with Sirt6 deletion (Yao et al., 2017). Decreased PGC-1α expression in brown fat was not attributed to changes in acetylation levels of H3K9 or H3K56 in its promoter region in Sirt6-deficient mice (Yao et al., 2017). Activating transcription factor 2 (ATF2) is recruited to the PGC-1α promoter after β-adrenergic receptor activation in BAT (Herzig et al., 2001; Cao et al., 2004). Sirt6 depletion reduced phosphorylated ATF2 binding to the PGC-1α promoter and subsequently decreased the thermogenic program of brown fat and led to obesity (Yao et al., 2017).

## Sirt6 and circadian rhythm

Circadian rhythm refers to changes in the life cycle during 24 h. Circadian rhythms play an important role in regulating body metabolism (Kohsaka et al., 2007; Barnea et al., 2009). In mice with circadian rhythm disruption, energy metabolism, especially glucose metabolism, was disturbed. Both behavioral and molecular circadian rhythms were changed in mice with metabolic disorders caused by an HFD (Kohsaka et al., 2007; Barnea et al., 2009). The clock has a central role in the circadian rhythm (Feng and Lazar, 2012). Bmal1 gene (also called aryl hydrocarbon receptor nuclear translocator-like [ARNTL]) is the central part of the biological clock transcription and translation feedback loop (Kiyohara et al., 2006). These two genes, encoding the protein together to form a Clock/Bmal1 isomer, play an important role in the feedback loop. Sirt6 is the only constitutive chromatin-associated sirtuin and is prominently present at transcriptionally active genomic loci (Masri et al., 2014). Sirt6 interacts with CLOCK/BMAL1 and, differently from SIRT1, governs their chromatin recruitment to gene promoters (Masri et al., 2014). Sirt6 contributes to chromatin recruitment of both the circadian machinery as well as SREBP-1 (Masri et al., 2014). Liver-specific deletion of Sirt6 downregulates hepatic rhythmic transcription, accompanied by changes in lipid metabolism (Masri et al., 2014). Deletion of Sirt6 leads to decreased binding of Clock/Bmal to the clock control gene (CCG) promoter and the binding of SREBP1 with its downstream gene promoters, thereby affecting lipid metabolism in the organism (Masri et al., 2014). The relationship between Sirt6 and fatty acid metabolism remains unclear. Sirt6 regulation of the circadian metabolic programs sheds new light on how the enzyme couples chromatin dynamics to metabolism (Masri et al., 2014; Tasselli et al., 2016b).

## Sirt6 and inflammation

Sirt6 deficiency increases the inflammatory response in many tissues. In adipose tissue, Sirt6 deficiency increases macrophage infiltration and adipose tissue inflammation and promotes HFD-induced insulin resistance (Kuang et al., 2017; Xiong et al., 2017). Sirt6 deficiency in mouse immune cells leads to liver inflammation and fibrosis (Xiao et al., 2012). In particular, in pancreatic cancer cells, Sirt6 induces the expression of proinflammatory cyto-/chemokines [interleukin 8 and tumor necrosis factor α (TNFα)] (Lappas, 2012). In endothelial cells, Sirt6 deficiency increased the expression of proinflammatory cytokines, extracellular matrix remodeling enzymes and adhesion molecules (Lappas, 2012). Loss of Sirt6 increased the expression of NF-κB, whereas overexpression of Sirt6 decreased NF-κB transcriptional activity (Lappas, 2012). Sirt6 interacts with the NF-κB subunit, deacetylates histone H3 lysine 9 (H3K9) at NF-kB target gene promoters, and inhibits the expression of downstream target genes, thus inhibiting the inflammatory reaction (Kawahara et al., 2009). In Sirt6-deficient cells, RELA promoter occupancy was increased; it enhanced hyperacetylation of NF-κB target gene promoters and induced NF-κB–dependent gene expression, cellular senescence and apoptosis (Kawahara et al., 2009). Sirt6 also binds to c-Jun and decreases the expression of its downstream target genes IL-6, monocyte chemoattractant protein 1, TNFα and H3K9, thereby inhibiting the expression of these genes (Xiao et al., 2012; Kuang et al., 2017).

## Sirt6 and DNA damage and diabetes

DNA damage is a permanent change of nucleotide sequence during DNA replication, resulting in the change of corresponding genetic characteristics (Shimizu et al., 2014). It is also related to the occurrence and development of chronic diseases, such as cancer and diabetes (Blasiak et al., 2004; Grindel et al., 2016). Sirt6 has been characterized as a histone deacetylase (HDAC) that targets specific sites (Michishita et al., 2008, 2009; Yang et al., 2009; Tasselli et al., 2016a). Sirt6 deacetylates the histone H3 on acetylated K9, K56 (Michishita et al., 2008, 2009) and the more recently identified K18 residue (Yang et al., 2009), causing the repression of many genes involved in inflammation, aging, genome stability, metabolic pathways and telomere integrity (Vitiello et al., 2017). Loss of Sirt6 affects the pathway of ATM/CHK2 and recruitment of repair factors to sites of DNA damage (Cagnetta et al., 2018). Sirt6 interacts, deacetylates and affects telomere repeat binding factor 2 (TRF2) stability, which may be part of a higher order complex with functional impacts on DNA damage response (DDR), cancer and aging (Rizzo et al., 2017). Moreover, following DNA damage, Sirt6 is recruited to double-strand breaks ensuring the proper activation of downstream DDR factors leading to an efficient DNA repair. Studies in diabetic patients showed greater oxidative damage to DNA. This indicates that the role of Sirt6 in DNA damage may as a new therapeutic pathway for cancer and diabetes related disorders.

## Conclusion

Excessive intake of carbohydrates or fat can lead to a range of metabolic syndromes, such as obesity, fatty liver and diabetes. With evidence that plays an important regulatory role in energy metabolism, it may be a potential therapeutic target for obesity and diabetes mellitus. Clinical trials investigating the use of sirtuin activators for treating diabetes are under way; such activators show promise as alternatives to current diabetes therapies. Thus, further research of sirtuin activators may result in a new class of safe, effective diabetes treatments.

## Author contributions

JK designed and wrote the manuscript. LC, QT, JZ, and YL contributed to the discussion and review of the manuscript. JH obtained funding and wrote the manuscript.

### Conflict of interest statement

The authors declare that the research was conducted in the absence of any commercial or financial relationships that could be construed as a potential conflict of interest.

## Abbreviations

ACC1: acetyl CoA carboxylase 1
ACOX1: acyl-coenzyme A oxidase 1
AMPK: AMP-activated protein kinase
ATF2: activating transcription factor 2
Bmal1: aryl Hydrocarbon Receptor Nuclear Translocator Like
C/EBPα/β/δ: CCAAT/enhancer-binding protein α, β, and δ
CCGs: clock control gene
CK2: enhancing casein kinase 2
CPT1α: carnitine palmitoyltransferase 1
DGAT1: diacylglycerol acyltransferase 1
FAS: fatty acid synthase
FoxO1: Forkhead box protein O1
G6p: glucose-6-phosphatase
GCN5: general control non-repressed protein 5
Glut1: glucose transporter 1
GPI: glucose-6-phosphate isomerase
HFD: high-fat diet
Hif-1α: hypoxia-inducible factor-1α
IGF-1: insulin-like growth factor 1
KIF5C: kinesin family member 5C
LDH: lactate dehydrogenase
PCSK9: proprotein convertase subtilisin/kexin type 9
PDK: dehydrogenase kinase
Pepck: phosphoenolpyruvate carboxykinase
PFK-1: phosphofructokinase-1
PGC-1α: peroxisome proliferator activated receptor γ coactivator 1-α
PGK1: phosphoglycerate kinase
PPARγ: peroxisome proliferator-activated receptor gamma
SCD1: stearoyl-CoA desaturase-1
SREBP 1/2: sterol regulatory element binding proteins 1/2
UCP1: uncoupling protein 1
ATGL: adipose triglyceride lipase
CR: calorie restriction
WAT: white adipose tissue
TG: triglycerides
LDL: low-density lipoprotein
TNFα: tumor necrosis factor α
IL-8: interleukin 8.
